# On-line daily plan optimization combined with a virtual couch shift procedure to address intrafraction motion in prostate magnetic resonance guided radiotherapy

**DOI:** 10.1016/j.phro.2021.07.010

**Published:** 2021-07-25

**Authors:** Daan M. de Muinck Keizer, Jochem R.N. van der Voort van Zyp, Eline N. de Groot-van Breugel, Bas W. Raaymakers, Jan J.W. Lagendijk, Hans C.J. de Boer

**Affiliations:** University Medical Center Utrecht, Department of Radiotherapy, 3508 GA Utrecht, the Netherlands

**Keywords:** Prostate cancer, Intrafraction motion, Soft-tissue registration, Plan adaptation, MR-guided radiotherapy, MR-Linac

## Abstract

**Background and purpose:**

In daily adaptive magnetic resonance (MR)-guided radiotherapy, plans are adapted based on the patient's daily anatomy. During this adaptation phase, prostate intrafraction motion (IM) can occur. The aim of this study was to investigate the efficacy of always applying a subsequent virtual couch shift (VCS) to counter IM that occurred during the daily contour and plan adaption (CPa) procedure.

**Material and Methods:**

One hundred fifty patients with low and intermediate risk prostate cancer were treated with 5x7.25 Gy fractions on a 1.5 T MR-Linac. In each fraction, contour adaptation and dose re-optimization was performed using the session’s first MR-scan. IM that occurred here was countered using two methods. One patient group had selective VCS (sVCS) applied if the CTV reached outside the PTV on a second MR acquired during plan optimization. The other group had always VCS (aVCS) applied for any prostate shift greater than 1 mm. Remaining IM during beam delivery was determined using 3D cine-MR.

**Results:**

Percentage of fractions where a VCS was applied was 28% (sVCS) vs 78% (aVCS). Always applying VCS significantly reduced influences of systematic prostate IM. Population random and systematic median values in all translations directions were lower for the aVCS than sVCS group, but not for the population random cranial-caudal direction.

**Conclusion:**

Applying VCS after daily CPa reduced impact of systematic prostate drift in especially the posterior and caudal translation direction. However, due to the continuous and stochastical nature of prostate IM, margin reduction below 4 mm requires fast intrafraction plan adaption methods.

## 1 Introduction

Hypofractionated magnetic resonance (MR)-guided radiotherapy for prostate cancer has become more common and is being used in different institutes. While the introduction of MR-guided radiotherapy allowed for more accurate treatment of prostate cancer patients [1], [2] the use of (ultra)-hypofractionated treatments also poses new challenges. Especially with extreme hypofractionation, the impact of intrafraction motion and thus the requirement for target motion management grows [3].

Currently, multiple machines are commercially available which combine magnetic resonance imaging with a linear accelerator [4], [5]. In MR-guided radiotherapy using daily adaptations, a daily MR-scan is obtained after which the plan is adapted to correspond with the actual anatomy [6], [7]. In such a workflow contours from simulation or pre-treatment scans are warped to the daily pre-treatment scan using a contour propagation algorithm. These warped contours are then adjusted where deemed necessary by attending radiation oncologists or radiotherapy technicians [8], [9].

Using online plan re-optimization in daily adaptive MR-guided radiotherapy for prostate cancer patients has been described by different groups [10], [11], [12], [13]. After the plan optimization phase, a second scan is acquired. This scan is referred to as the position verification (PV) scan, and used to assess if no large intrafraction motion occurred during the optimization phase. When large anatomical changes have occurred, (e.g. bladder filling and/or rectal gas pockets), options include to reposition the patient or to repeat the online plan re-optimization step with contour adjusting.

An important issue with online plan re-optimization procedures is the time it requires to perform. The daily contour adaptation procedure and plan re-optimization combined can require more than 15 min, during which intrafraction motion can occur [14]. To counter for such motion, strictly treatment planning margins cannot be reduced below 5 mm without the ability to further modify the plan during radiation delivery [15]. In extreme hypofractionation such a margin reduction is desired.

Besides patient repositioning, a virtual couch shift (VCS) can be applied to the planned dose [16]. This procedure allows plan adaptation based on the patient's position. Effectively, the PV scan is rigidly registered to the daily pre-treatment scan after which the reference data isocenter is updated [8]. Then, plan re-optimization is started using the pre-treatment MR-scan and contours. With this approach, contour adaptation is thus not performed or possible as plan re-optimization is based on the original contours. The VCS method can be used to correct for intrafraction motion which has occurred during the plan optimization phase. Menten et al. [17] reported a study on five patients, where online plan re-optimization was applied and followed by VCS when the clinical target volume (CTV) was outside the planned target volume (PTV) on the PV scan. A similar approach for prostate patients was described by our group [14] as well as in a study on bladder cancer patients [18]. In this study, we investigate the efficacy of always applying the VCS procedure for any prostate shift over 1 mm to counter the intrafraction motion which occurred during the online plan re-optimization phase.

## 2 Material and methods

One hundred fifty (1 5 0) patients with low to intermediate risk prostate cancer (NCNN classification) were registered as part of an institutional review board approved registration and imaging study. All patients were treated on a 1.5 T MR-Linac (Elekta Unity) and underwent hypofractionated prostate radiotherapy with five fractions of 7.25 Gy delivered in 2.5 weeks between July 2019 and September 2020 at the University Medical Center Utrecht.

### 2.1 Workflow

During each fraction, an initial T2 weighted 3D MR scan (Pre) was obtained. This sequence had a 2-minute scan time, a dimension of 480 × 480 × 300 voxels and a voxel spacing of 0.83 × 0.83 × 1.0 mm^3^ Planning MRI contours were automatically propagated and manually adapted to this Pre-scan. Thereafter, full plan re-optimization was started in the Monaco MR-Linac treatment planning systems (TPS) (v5.40.00 and later v5.40.01) using the online plan re-optimization (‘Adapt to Shape’ or ATS) workflow [8].

As the average time between the Pre scan and beam-on is approximately 27 min, prostate intrafraction motion has to be taken into account [14]. Therefore, during the last minutes of the ATS process, a position verification (PV) scan was obtained. This PV scan was acquired with the same scan parameters as the Pre scan and used to determine the prostate intrafraction motion during the ATS process.

Two groups were defined in this study. The selective virtual couch shift (known in the 1.5 T MR-Linac workflow as ‘Adapt to position’ or ‘ATP’) group (sATP) included 26 patients who had treatment between July 2019 and January 2020. In this group, the ATP procedure was applied if part of the prostate was outside the PTV as judged visually on the PV scan. Influences of beam alignment errors, image distortions or delineation inaccuracies were neglected, as these errors are on a sub-millimeter scale [6], [19]. In the ATP procedure standard segment weight optimization is used. The ATP translation was obtained from a registration using a clipbox around the prostate and adjusted manually if necessary.

The always ATP group (aATP) consisted of 124 patients, who had treatment between January 2020 and September 2020. In this group, the ATP procedure was applied for any prostate shift greater than 1 mm. This procedure was performed in an attempt to further reduce the impact of prostate intrafraction motion.

For all patients, a 7-beam intensity modulated radiotherapy (IMRT) technique was applied with a 5 mm CTV to PTV margin. During plan delivery 3D cine-MR dynamics were acquired every 9.4 s. Technical details of the cine-MR sequence and the workflow with steps and timings are provided in the Supplementary material and Fig. S1. Details on the ATP and ATS procedure for the 1.5 T MR-Linac were described by Winkel et al [8].

### 2.2 Registration

An independent prostate rigid registration (IPR) method was used to determine the prostate intrafraction motion on all cine-MR dynamics [20]. This IPR-method was also used to rigidly register the first cine-MR dynamic to the Pre and PV scan, as well as to register the PV to the Pre scan. These results combined were used to determine the prostate intrafraction motion during the complete course of MR-guided radiotherapy sessions. This approach was previously described and the complete course is defined as the period in which the patient is positioned on the treatment table (~45 min) [14]. Importantly, the IPR-method uses translations and rotations, while the ATP method in the TPS is solely translation based.

The ATP shift performed in the TPS was extracted from exported dicom plan files. This ATP shift was then compared to the IPR-method based rigid registration result obtained between the PV and Pre scan. All timings reported in this study were extracted from dicom headers.

To determine the actual effect of applying ATP, the reported intrafraction motion results were calculated from cine-MR dynamics with respect to the Pre scan. The ATP shift applied in the TPS was then subtracted from this value. We had to apply this 2-stage approach because the ATP correction does not perfectly describe the shift from Pre to PV scan, as the underlying Monaco TPS registration method has a finite accuracy and is limited to translations only. Therefore, direct registration of cine-MR dynamics to the PV scan would misrepresent the actually applied correction. As the ATP procedure only corrects for translational motion, all intrafraction rotation as determined from the cine-MR dynamics is plotted with respect to the Pre scan without further adjustments*.*

### 2.3 Statistical analysis

ATP shifts applied in the TPS were compared to the obtained translational registration result between the PV to PRE scan with the IPR method.

Both the population systematic (Σ) and population random (σ) error were calculated. Determining these errors was described in our previous study on fiducial marker tracking in 3D cine-MR [21]. The population systematic error describes the mean displacements spread over the group of patients at any time point. The population random error expresses the corresponding interfraction fluctuation with respect to these patient-specific means [22]. The results from the current study will be compared to our previously reported population random and systematic errors. For comparison purposes, the group from this previous study with fiducial marker tracking is referred to as “FMT-group''.

Wilcoxon rank sum tests were performed to test significant population median differences between the sATP and aATP groups as described in the Supplementary material.

## 3 Results

Overall, 28% of the fractions in the sATP group required an ATP step, while 78% of the fractions in the aATP group had ATP applied. Timings required for plan adaptation and treatment of both groups are provided in Table 1. The mean time between the PV and beam-on was 5 min, but as the start of this sequence was a manual step, fluctuations were present. Time required for actual ATP dose calculation (VCS) was below 1 min.

**Table 1:** Overview of timings, plan adaptation is based on the time between the end of the Pre scan and the start of the first cine-MR acquisition, thus until the start of the beam-on period. Total fraction time is based on the acquisition time of the last cine-MR dynamic with respect to the Pre scan for each fraction. The reported n-number shows the included number of fractions.

	Plan adaptation Mean time ± one std. (time in minutes)	Total fraction time mean time ± one std. (time in minutes)
Selective ATP		
without ATP applied (n = 97)	28.8 (±7.1)	40.1 (±7.3)
Selective ATP		
with ATP applied (n = 30)	33.2 (±5.7)	45.4 (±6.2)
Always ATP		
Without ATP applied (n = 131)	26.9 (±7.3)	38.5 (±7.6)
Always ATP		
With ATP applied (n = 459)	26.7 (±5.4)	38.6 (±5.6)

**Fig. 1:**
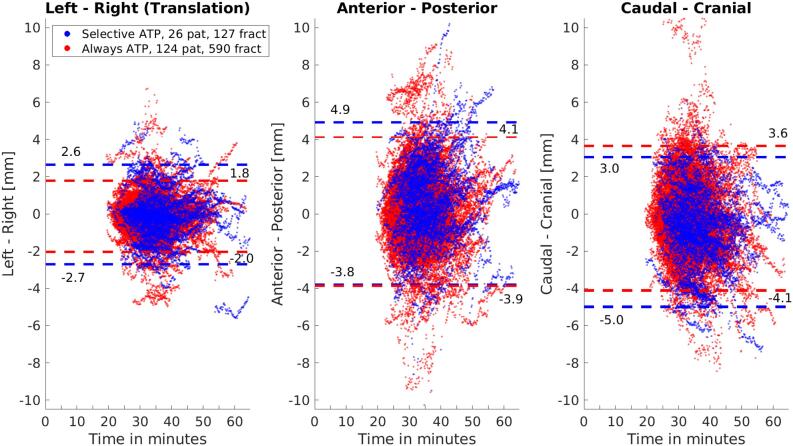
Prostate intrafraction translation graphs for all patients with respect to the daily pre-treatment scan. The horizontal lines provide the 95 percentile confidence intervals.

**Fig. 2:**
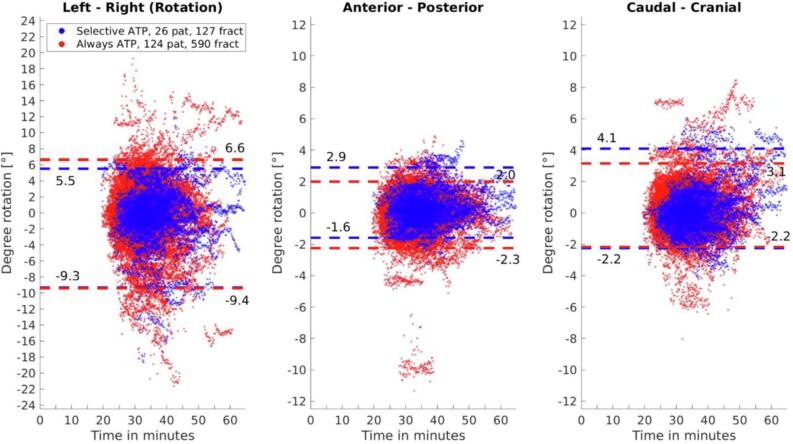
Prostate intrafraction rotation graphs for all patients with respect to the daily pre-treatment scan. The horizontal lines provide the 95 percentile confidence intervals.

The largest 95 percentile confidence intervals for prostate intrafraction motion were found in the posterior (sATP: 4.9 mm vs aATP: 4.1 mm) and caudal (sATP: −5.0 mm vs aATP: −4.1 mm) translation directions (Fig. 1). Moreover, significant rotations about the left–right (LR) rotation axis were present, with the 95 percentile confidence interval for both groups reaching over −9 degrees (Fig. 2). In these graphs, then intrafraction motion as found using 3D cine-MR imaging during beam-on was plotted with respect to the daily Pre-scan. Fractions in which an ATP shift was applied were corrected for as described previously.

**Fig. 3:**
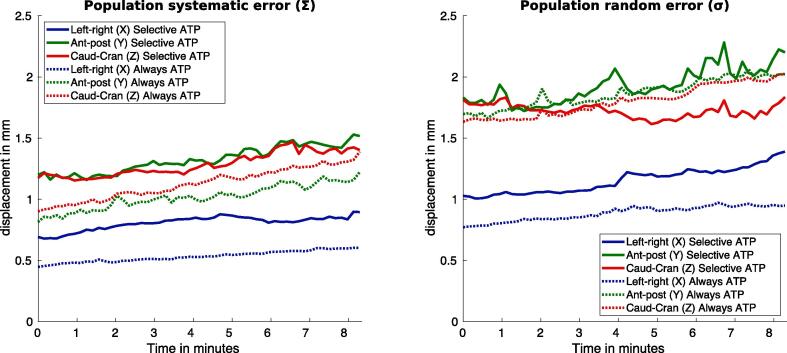
The population systematic errors (Σ, provided on the left-hand side) and population random errors (σ, provided on the right-hand side) over time, for the tree main directions and both groups. The errors are provided with respect to the daily pre-treatment scan and incorporate applied ATP shifts.

Population systematic errors (Σ) in the aATP group were smaller than found for the sATP group (Fig. 3). The population random (σ) left–right (LR) error for the aATP group was smaller than seen in the sATP group, while this was not the case for the anterior-posterior (AP) and caudal-cranial (CC) random errors. The graphs presented here are provided with respect to the daily pre-treatment scan and incorporate applied ATP shifts. Population systematic and random errors during the beam-on period with respect to the first cine-MR dynamic are provided in Fig. S2 of the Supplementary Material.

Results from the Wilcoxon rank sum test showed that the population random and systematic median values in all translations directions were lower for the aATP than sATP group, but not for the population random cranial-caudal (Z) direction. These results are provided in Table 2.

**Table 2:** Overview of the Wilcoxon rank sum test results, over the population systematic (Σ) and population random (σ) errors of the sATP and aATP group.

Translation direction	Population systematic error (Σ)	Population random (σ)
X (left–right)	Null hypothesis rejected (p < 1e−5)	Null hypothesis rejected (p < 1e−5)
Y (anterior-posterior)	Null hypothesis rejected (p < 1e−5)	Null hypothesis rejected (p = 0.02)
Z (caudal-cranial)	Null hypothesis rejected (p < 1e−5)	Null hypothesis NOT rejected (p = 0.99)

Correlation coefficients between the clipbox match as performed in the TPS, versus the IPR-method were 0.8 for LR, 0.9 for AP and 0.8 in the CC translation direction with all p-values below 1e-5. The mean difference and standard deviation between the IPR-method and Monaco match was 0.0 ± 0.7 mm in LR, 0.1 ± 1.2 mm for AP and 0.2 ± 1.4 mm in the CC translation direction. Mean-difference plots are provided in Fig. S3 in the Supplementary Material.

## 4 Discussion

In this work the efficacy of applying a VCS to counter prostate intrafraction motion that occurred during the daily contour and plan adaption procedure was studied. Applying a VCS after daily contour and plan adaption has shown to reduce the impact of systematic prostate drift in especially the posterior and caudal translation direction.

While the always ATP group involved a more elaborate workflow than in the selective ATP group, an overall shorter mean total fraction time was found (Table 1). The effect may be attributed to a learning curve of the treatment personnel, with fraction timings becoming slightly shorter with more fractions performed. The average time between the PV scan end and beam-on was 5 min, but large fluctuations were present with outliers exceeding 10 min. As this scan was started manually prior to the end of the plan re-optimization, human judgment and ATS plan optimization time fluctuations influenced these timings. Nevertheless, a 45 min time slot was appropriate for all patient fractions.

Fig. 1 showed that the 95 percentile confidence intervals of the aATP group in the left–right, posterior and caudal translation direction with respect to the sATP group were reduced. However, this was not the case in the cranial translation direction. Applying ATP negated the impact of prostate drift in the caudal direction, which resulted in a slightly larger 95 percentile confidence interval in the cranial direction as the variance in the cranial direction was no longer dampened by the overall prostate drift in the caudal direction.

Some outliers worth noticing were visualized as the sATP group trace, with a LR translation value of −5 mm at the timepoint of 55 min. Here, an error of 4 mm was introduced due to manual Monaco clipbox match adjustment. For both groups, even larger outliers as an effect of unstable gas pockets with a tendency to push the prostate can be seen in the anterior and cranial translation direction. Similar effects of gas pockets on prostate intrafraction motion were reported in our previous study on seminal vesicle tracking [23] and in literature [24], [25]. Obviously, the present ATP procedure cannot correct for such sudden events.

As the applied ATP correction only includes translation, the procedure had no effect on intrafraction rotation between both groups. This effect is visualized in Fig. 2, in which no significantly reduced aATP group 95 percentile confidence interval with respect to the sATP group was seen. In this graph, the larger negative 95 percentile confidence interval about the left–right rotation axis stands out. In cases with large gas pockets, significant (negative) left–right rotation was observed and the negative outliers in this graph were all cases impacted by gas pockets in the rectum. Interestingly, scenarios with intrafraction rotation due to drift as a result of anatomical sagging (e.g. patient relaxation) normally tend to result in positive left–right rotation values. However, the significant impact of gas pockets on the rotational values seemed to outweigh the rotation motion caused by drift, resulting in an overall negative intrafraction rotation trend. With only rotation about the left–right rotation axis the CTV (prostate corpus) may stay in the PTV. However, the effect of rotation about the left–right rotation axis was significantly larger if seminal vesicles were also (partially) included in the CTV [23].

In our previous study including 50 patients we found drift to be the major intrafraction motion component in 30% of all fractions and during at least one fraction in 76% of all patients [23]. Intrafraction motion mainly caused by gas pockets was observed in 18% of all fractions and occurred in at least one fraction in 50% of all patients.

The “always ATP“ method reduced the median population random and systematic median values in all translations directions compared to the sATP group, but not for the population random cranial-caudal (Z) direction as proven with the Wilcoxon rank sum test (Table 2) and presented in Fig. 3. The effect of gravitational sagging and bladder filling on the prostate intrafraction motion were reduced by applying the ATP procedure. The effect of gravitational sagging and bladder filling on the prostate intrafraction motion was reported in previous studies [15], [26]. Although reducing margins based on using the always ATP method alone is debatable, applying ATP after ATS had a beneficial impact by reducing the bladder volume in the high dose region.

Previously we also reported systematic and random errors for 29 patients [21]. Importantly, in this previous study the patients were positioned on the treatment table for a short time period ( 2 min) before the cine-MR imaging start. To compare both studies, we included a graph with the population systematic and random errors with respect to the first cine-MR dynamic in the Supplementary Material (Fig. S2). This approach was similar to our previous study [21].

In the first 4 min during beam-on, the population systematic error results showed similar trends for all groups (sATP, aATP and the FMT-group). Toward the end of the beam-on period (after 7 min), the sATP and aATP group tend to show smaller increases than found in the FMT-group. The patients in the sATP and aATP group were positioned significantly longer on the treatment table before the cine-MR image acquisition start (27 min). This longer on-couch period may potentially have led to intrafraction motion saturation, as the prostate is limited by anatomical boundaries.

Similar population random error trends were seen for all three groups over the complete beam-on period and no difference was found which may be explained by the difference in the on-couch period. This effect underlines the fact that prostate intrafraction motion on a population base follows a random walk model, as described by Ballhausen et al. [27]. Applying the ATP shift thus reduced the influences of systematic prostate intrafraction motion, but had little impact on the random motion component. When considering the impact of intrafraction motion during a single fraction for an individual patient, intrafraction motion behavior can be much more irregular (Fig. 1). To reduce the impact of this random motion component, fast re-optimization schedules are required based on fast 3D cine-MR imaging data such as described by Kontaxis et al. [28], [29].

The intrafraction motion between the Pre and PV scan as found with the Monaco TPS was compared to the IPR-method (Fig. S3). Overall, good correlations were found, with the highest correlation in the AP direction (R = 0.9). In our opinion, the clipbox match can be used to effectively apply ATP but only after sufficient training in manual adjustment. Details on the accuracy of the ATP registration is provided in the Supplementary Material.

To conclude, always applying ATP before dose delivery reduced the impact of systematic prostate intrafraction motion. The effect of prostate drift in the posterior and caudal translation direction was reduced allowing for potentially slightly better sparing of bladder wall. However, due to the continuous and stochastical nature of intrafraction motion, margin reduction below 4 mm is not advised and would require fast intrafraction plan adaptation methods, particularly if the number of fractions is reduced further.

## Declaration of Competing Interest

The authors declare that they have no known competing financial interests or personal relationships that could have appeared to influence the work reported in this paper.
