# Supplementary material for: On-line daily plan optimization combined with a virtual couch shift procedure to address intrafraction motion in prostate magnetic resonance guided radiotherapy
Source: Phys Imaging Radiat Oncol. 2021 Jul 25;19:90–5. doi: 10.1016/j.phro.2021.07.010 (PMC8327343; doi:10.1016/j.phro.2021.07.010)
Supplement: Supplementary data 1 [file mmc1.docx]

**Supplementary Material**

During plan delivery, 3D cine-MR dynamics were simultaneously acquired using a balanced turbo field echo sequence. Each 3D dynamic spanned 9.4 seconds, and consisted of 448×448×45 reconstructed voxels with a voxel spacing of 1.0×1.0×2.2 mm^3^.

The 1.5T MR-Linac workflow with steps and approximate cumulative timings is visualized in figure S1.


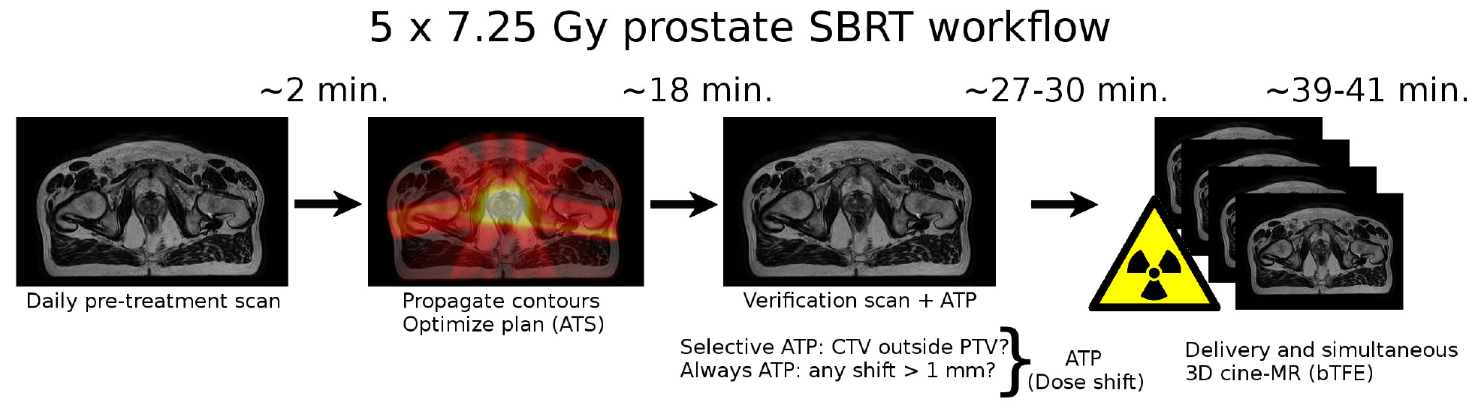


Figure S1: Schematic overview of the workflow for prostate SBRT on the 1.5T MR-Linac. Approximate cumulative timings are provided in the figure.

The Wilcoxon rank sum test is a nonparametric test for equality of population medians and used to determine whether or not the median population systematic and median population random values of the aATP group were significantly lower than the median values of the population systematic and population random values of the sATP group. Tailed analysis was used with a significance level of 5%, where the null hypothesis states that the data of both groups are samples from continuous distributions with equal medians and where the alternative hypothesis states that the median population systematic or median population random error of the sATP group is larger than the median population systematic or median population random error of the aATP group. While the applied statistical test is a non-parametric test for unequal sized groups, it is important to note that the aATP group is significantly larger than the sATP group, and the aATP study started after RTTs had gained practical experience with sATP.


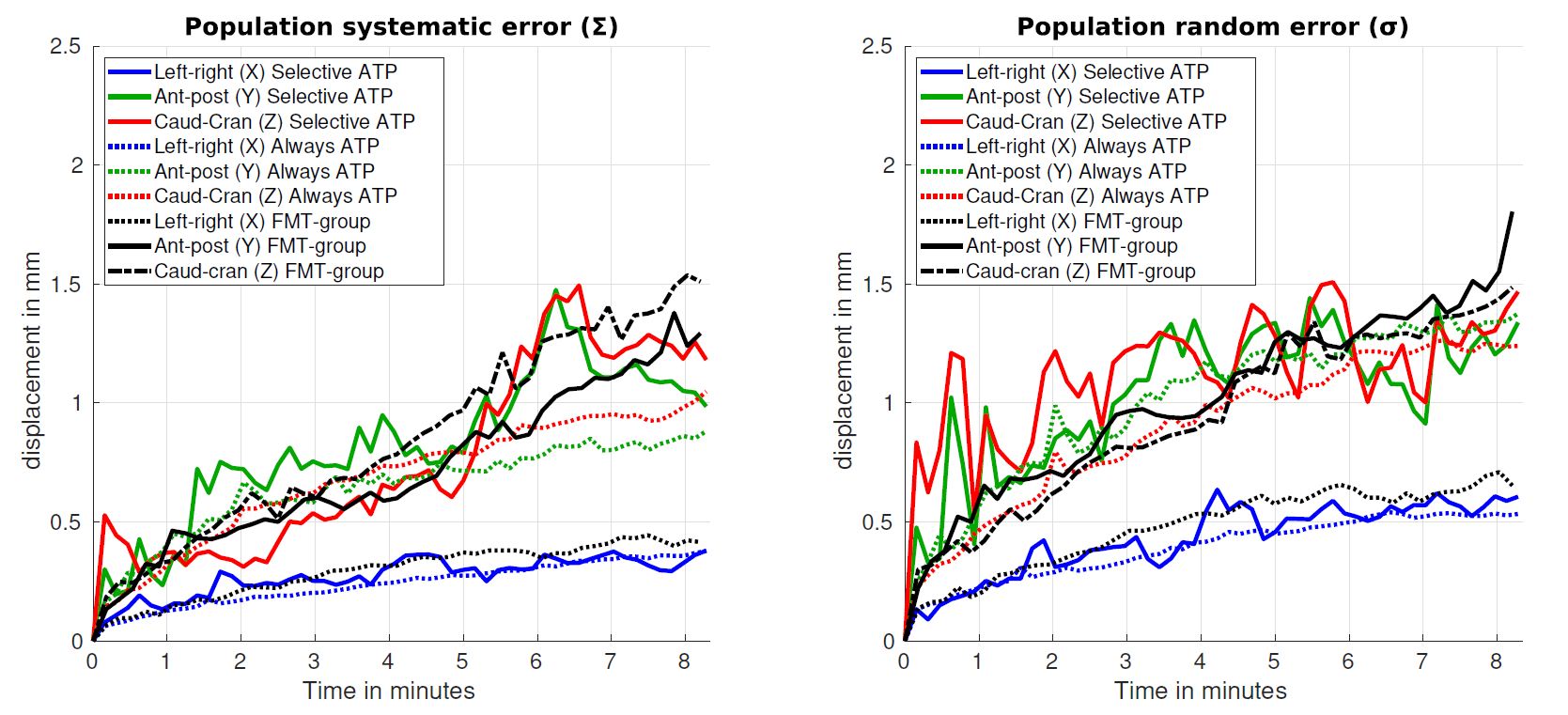
Figure S2 provides both the population systematic and random errors, in which all data points are plotted with respect to the first cine-MR dynamic. The data from the fiducial marker tracking group (FMT-group) is also included in these graphs.

Figure S2: The population systematic errors (Σ, provided on the left-hand side) and population random errors (σ, graph on the right-hand side) over time, for the tree main directions and all groups. All points are plotted with respect to the first cine-MR dynamic, at time point zero.

Mean-difference (Bland-Altman) plots for the ATP match as established from clipbox matching in the treatment planning system (TPS), versus the independent prostate registration (IPR) method are provided in figure S3. Our experience with the ATP procedure is that it nearly always creates the desired virtual couch shift, i.e. it shifts the ATS dose distribution nearly perfectly. In rare occasions this shift may not be completely perfect, which may result in some PTV coverage reduction of 1-2% but this is in our opinion not clinically significant: the correction for intrafraction motion is more important than the potential slight coverage loss.

The Monaco ATP match could be improved by incorporating rotations for the registration part as well as mask-based instead of clipbox-based rotations. Incorporating rotations for the ATP match would however generate values that could not be corrected with the ATP approach, for which additional solutions may be required, such as segment aperture morphing. During this study, beam-gating on the MR-Linac was not yet technically implemented in a clinical setting. Therefore, no action was undertaken for any intrafraction motion occurring during the beam-on period.

A larger outlier is found in the CC translation direction with an IPR-method value of 5 mm, versus about -6 mm in Monaco (figure S3). This error was caused by erroneous manual ATP registration adjustment in the TPS. Moreover, voxel size discretization effects can be observed especially in the CC translation direction. The right panel of figure S3 shows Monaco ATP shifts of 0 mm, while values found with the IPR-method range from -4 to 2 mm. The apparent voxel discretization effect may be caused by the underlying registration implementation in Monaco. However, further research in this topic is required. Registration result differences may also be caused by the fact that a square clipbox enclosing the prostate was used in Monaco, which included parts of surrounding anatomy. For the IPR-method, only the prostate body (CTV) was used, without surrounding anatomy. Moreover, the IPR-method incorporates rotations, whereas the Monaco method is solely translation based. Incorporating both translations and rotations, in addition to only using the prostate CTV as registration mask results in registrations with a more accurate match. Still, the results in figure S3 show that the clipbox match as performed in the Monaco TPS is of in general sufficient quality especially after appropriate training.


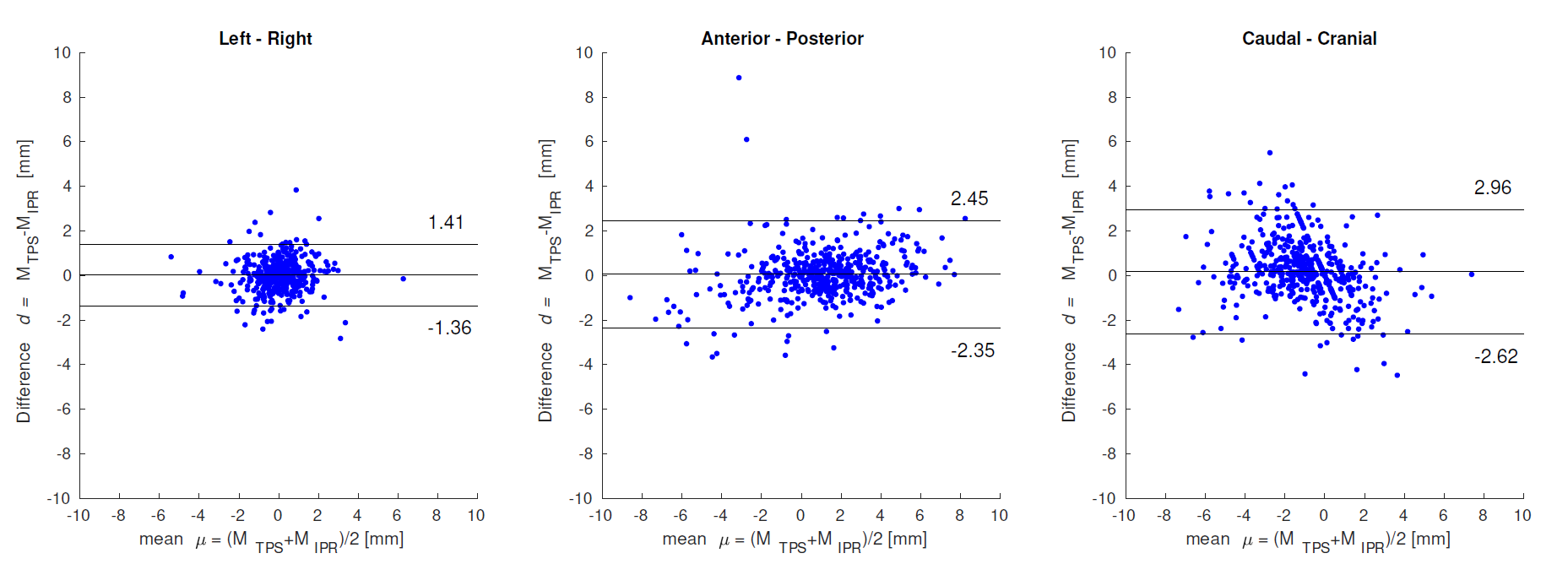


Figure S3: Mean-difference plots for the ATP match as established from clipbox matching in the treatment planning system (TPS), versus the independent prostate registration (IPR) method. Manual shift adjustments made to the TPS clipbox registrations are included in these data. In these graphs the available data points of 488 fractions over 138 patients are included. The three horizontal lines show the upper 95% limit of agreement, group mean and lower 95% limit of agreement.
